# Identification of Lysine 37 of Histone H2B as a Novel Site of Methylation

**DOI:** 10.1371/journal.pone.0016244

**Published:** 2011-01-13

**Authors:** Kathryn E. Gardner, Li Zhou, Michael A. Parra, Xian Chen, Brian D. Strahl

**Affiliations:** Department of Biochemistry and Biophysics, School of Medicine, Lineberger Comprehensive Cancer Center, University of North Carolina, Chapel Hill, North Carolina, United States of America; Texas A&M University, United States of America

## Abstract

Recent technological advancements have allowed for highly-sophisticated mass spectrometry-based studies of the histone code, which predicts that combinations of post-translational modifications (PTMs) on histone proteins result in defined biological outcomes mediated by effector proteins that recognize such marks. While significant progress has been made in the identification and characterization of histone PTMs, a full appreciation of the complexity of the histone code will require a complete understanding of all the modifications that putatively contribute to it. Here, using the top-down mass spectrometry approach for identifying PTMs on full-length histones, we report that lysine 37 of histone H2B is dimethylated in the budding yeast *Saccharomyces cerevisiae*. By generating a modification-specific antibody and yeast strains that harbor mutations in the putative site of methylation, we provide evidence that this mark exist *in vivo*. Importantly, we show that this lysine residue is highly conserved through evolution, and provide evidence that this methylation event also occurs in higher eukaryotes. By identifying a novel site of histone methylation, this study adds to our overall understanding of the complex number of histone modifications that contribute to chromatin function.

## Introduction

In eukaryotic cells, DNA is packaged in the form of chromatin. Approximately 147 base pairs of DNA wrap around an octomer composed of two H2A–H2B dimers and one H3–H4 tetramer to form nucleosomes, the fundamental repeating unit of chromatin [1], [2]. Because nucleosomes are organized into progressively higher-ordered structures, significant chromatin remodeling is necessary for the numerous DNA-templated processes that must occur for normal cellular function, such as transcription, DNA replication, DNA repair, and chromosome segregation.

One means by which alterations to chromatin structure is accomplished is through post-translational modifications (PTMs) of the histone proteins. The core histones are largely globular, with the exception of unstructured N-terminal tails that protrude from the surface of the core particle. Although numerous PTMs have been shown to occur on residues located on the histone tails [3], it is becoming increasingly evident that residues within the globular domain are also subject to modifications [4], [5], [6]. The type of PTMs demonstrated to occur on histone proteins include acetylation, methylation, phosphorylation, ubiquitylation, sumoylation, ADP ribosylation, proline isomerization, citrullination, butyrylation, propionylation and glycosylation [3], [7], [8]. While the functional significance of some of the aforementioned modifications remains to be elucidated, it is well established that other histone PTMs function by at least one of the following mechanisms: (1) disruption of nucleosomal contacts between histones and their associated DNA or between histones in contiguous nucleosomes, or (2) recruitment of non-histone proteins [3], [4]. Acetylation of lysine residues is the best-characterized modification shown to affect higher-order chromatin structure, where this mark neutralizes the basic charge of the residue on which it occurs, thereby inhibiting histone-histone or histone-DNA interaction and thus chromatin compaction [9], [10], [11]. With regard to the other means by which histone PTMs can function, the recruitment of non-histone proteins is facilitated by the ability of specialized domains to recognize and bind to defined marks [12]. For example, methylation of specific lysine residues in a defined state (mono-, di-, or trimethyl) can serve as a binding platform for effector proteins containing one of the following types of methyl-binding domains: chromodomain, tudor domain, PHD finger, MBT, Ankyrin repeat, PWWP domain and WD40 repeats [12], [13], [14].

The complexity of the number and diverse types of PTMs has led to the hypothesis of a “histone code” [15], [16], which posits that combinatorial patterns of histone PTMs lead to defined biological outcomes brought about by the recruitment of effector proteins necessary for function in DNA-templated processes. For example, TAF1 (the largest subunit of the TFIID complex which is involved in initiating the assembly of transcriptional machinery) contains a double bromodomain that preferentially binds to multiply acetylated histone H4 [17], and itself can function as a histone acetyltransferase [18]. There are numerous other examples of how defined combinations of histone modifications positively or negatively affect recruitment of specific proteins [19], [20], [21], [22], [23], [24]. Despite the identification of numerous histone PTMs to date, it is likely that other modifications still await discovery. Thus, of immediate importance in deciphering the histone code is the need for identifying all the PTMs that are present on histones, so that subsequent studies can be completed to determine the combinatorial patterns in which such modifications exist on physiological substrates and what the functional outcomes of such combinations are.

In recent history, mass spectrometry (MS) has widely been used as the primary method to identify histone PTMs. MS studies have commonly employed the bottom-up approach, in which short peptides derived from proteolytic cleavage of reverse-phase HPLC (RP-HPLC)-purified histones are analyzed by MS with peptide mass fingerprinting (PMF) or a combination of liquid chromatography (LC) and tandem MS (MS/MS) using electron transfer or collision-induced dissociation methods (ETD and CID, respectively) [25]. While this technique is a highly effective means by which to determine the molecular mass (by MS-PMF) or the sequence of a protein (by LC-MS/MS), it is limited in that incomplete sequence coverage of the protein of interest often occurs, and proteins with multiple cleavage sites (including the histone core proteins, which are rich in lysine and arginine residues) result in peptide segments that are too small for effective retention and/or detection [26], [27], [28], [29], [30]. More recently, advances in MS have led to the development of the top-down approach as a complementary method to bottom-up analysis as a highly useful means by which to identify PTMs on histones [31], [32], [33], [34], [35], [36], [37]. Full-length proteins are analyzed with top-down MS, as samples are infused into the mass spectrometer by electrospray ionization (ESI), allowing for MS/MS fragmentation via ETD or electron capture dissociation (ECD) of intact proteins [25]. A major advantage of top-down MS is that combinatorial patterns of modifications that exist on a single histone molecule can be identified [38], which is particularly valuable in outlining the global landscape of PTMs on histone proteins.

In this study, we sought to use top-down MS to analyze the global landscape of PTMs on histone H2B. From this analysis, we identified lysine 37 of histone H2B (H2BK37) as a novel site of methylation in the budding yeast *Saccharomyces cerevisiae*, and that this modification exists in the dimethyl state. We generated an antibody specific for dimethylated H2BK37 (H2BK37me2), with which we were able to confirm that this mark does in fact occur *in vivo*. Though our candidate approach to identify the methyltransferase responsible for placing this mark and phenotypic analysis to reveal a biological function did not offer conclusive results, we provide evidence that this modification is evolutionarily conserved supporting its overall importance as a novel histone modification. Furthermore, these results demonstrate that despite the numerous rounds of previous MS analysis, additional series of MS analyses employing recent technological advancements are necessary for continued identification of novel sites of modifications to generate a more complete atlas of the factors that putatively function in the context of the histone code.

## Results

### H2B is dimethylated at lysine 37

To date, only three lysine residues have been well-characterized as sites of methylation in budding yeast (namely lysines 4, 36, and 79 of histone H3) [39]. In higher eukaryotes, methylation is known to also occur on histone H3 at lysine residues 9 and 27 and histone H4 at lysine 20 [40]. To begin to address whether histone methylation occurs on other sites in budding yeast, as well as to acquire a more comprehensive atlas of histone PTMs, we sought to use MS analysis to identify novel histone modifications. Given recent advancements in MS technology, it is now possible to use the top-down MS approach to analyze intact histone proteins, thereby allowing for more precise delineation and quantification of the complex modified forms in which the histones exist [25]. We initially performed our top-down MS studies on histone H2B, as this histone has more recently been shown to be monomethylated at lysine 5 in humans [41], [42], and we were interested in determining whether this modification is conserved or if alternative sites of methylation exist in budding yeast.

**Figure 1:**
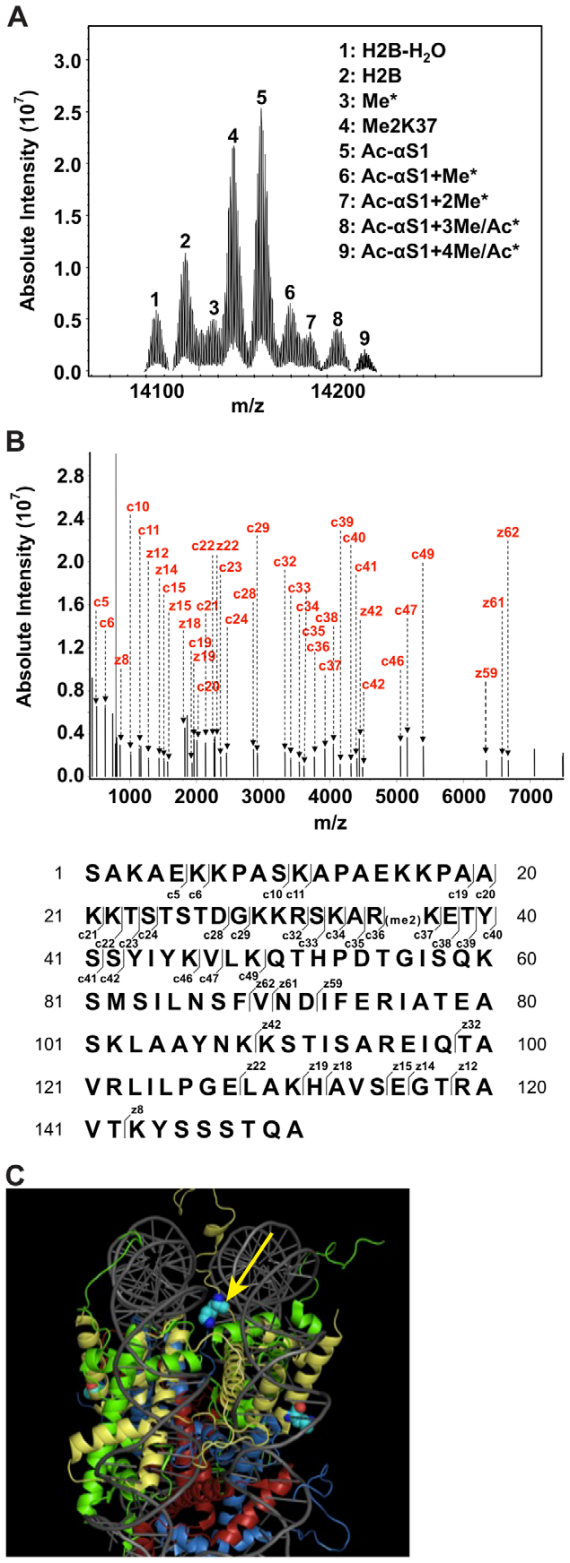
Top-down mass spectrometry (MS) analysis reveals histone H2B is dimethylated at lysine 37. (**A**) Top-down μESI-FTICR-MS analysis of yeast histone H2B. Shown is a mass spectrum of H2B revealing multiply modified forms of this histone, as indicated by peaks numbered 1–9. Each peak was analyzed by top-down μESI-FTICR-MS/MS analysis and modifications identified are denoted in the legend. Asterisks indicate PTMs that were not assigned. 100 scans per spectrum were acquired in the ICR cell with a resolution of 580,000 at m/z 400 Da. (**B**) Top-down μESI-FTICR-MS/MS analysis of peak 4. ECD MS/MS spectrum of histone H2B with two methyl marks (precursor: m/z 1415.9 Da, 10+ charge state) reveals lysine 37 is dimethylated. N-terminal (c ions) and C-terminal (z ions) fragment ions are assigned and shown in the upper panel. Lower panel denotes the ions in the sequence. Unassigned ions are either internal fragment ions or electronic noise. 100 scans per spectrum were acquired in the ICR cell with a resolution of 580,000 at m/z 400 Da. (**C**) Lysine 37 of H2B is located within the DNA gyres in the nucleosomal structure. Histones H2A, H2B, H3 and H4 are shaded green, yellow, red, and blue, respectively. The DNA backbone is colored gray. The yellow arrow points to the location of lysine 37 of histone H2B. The nucleosomal representation was generated using open-source PyMOL software (PyMOL 0.99rev10, DeLan Scientific LCC) with structural data taken from [100] (PDB file 1kx5).

According to its amino acid sequence, the theoretical monoisotopic mass ([M+H]^−^) of yeast histone H2B is 14113.6056 Da. Using a 12 Tesla Bruker Daltonics μESI-FTICR-MS with ultrahigh mass accuracy and resolution, exact mass measurement of the protein was performed to validate sample preparation of histone H2B following isolation from yeast nuclei and RP-HPLC purification. The experimental monoisotopic mass of one of the major peaks (peak 2) was at 14113.6028 Da, extremely close to the theoretical value (mass error<1 ppm) (Figure 1A). Patterns of PTMs of yeast histone H2B were also mapped by exact mass measurement. The PTM site(s) on each form was further identified and characterized based on exact masses and sequence information from MS and MS/MS experiments. Relative abundances of modified forms were obtained by integrating the four most abundant isotopic peaks in three different charge states of MS spectra and taking their sum (Table 1).

**Table 1:** Yeast histone H2B patterns of PTMs.

Yeast H2B PTM	Relative Abundance* (%)
H2B-H_2_O	7.0
H2B	12.8
Me**	5.5
Me2K37	25.7
Ac-αS1	29.9
Ac-αS1+Me**	7.3
Ac-αS1+2Me**	4.7
Ac-αS1+3Me/Ac**	4.6
Ac-αS1+4Me/Ac**	2.4

*RSD = ±1.0%.

**PTM sites cannot be assigned.

With a mass of 14141.6352 Da, the second strongest peak (peak 4) exactly matched the theoretical monoisotopic mass of yeast histone H2B with two methyl marks (mass error<1 ppm). To identify the modification site(s), the precursor ion corresponding to the modified protein (m/z 1415.9 Da, 10+ charge state) was isolated for top-down experiments using μESI-FTICR-MS with ECD (Figure 1B, upper panel). Inspection of the c and z fragment ions derived from the ECD MS/MS spectrum revealed +28 Da mass shifts of c_37_ to c_49_ ions, indicating that lysine 37 is dimethylated (Figure 1B, lower panel). As indicated in Table 1, the relative abundance of dimethylated lysine 37 on histone H2B is over 25.7% in all yeast protein isoforms. Other PTMs (e.g., sites of acetylation and methylation) could be identified based on ECD MS/MS experiments. However, with the exception of N-terminal acetylation at serine 1 (*data not shown*), which has previously been identified [29], [43], [44], additional PTMs could not be conclusively assigned.

The finding that lysine 37 of histone H2B is dimethylated is in agreement with recently published MS results from a study surveying for sites of lysine propionylation and butylyration [45]. However, very little is known about this lysine residue. Physically, lysine 37 of histone H2B is located between the DNA gyres of the nucleosome structure (Figure 1C). A previous study surveying the role of the N-terminal domain of histone H2B in transcription on a genome-wide level demonstrated that residues 30–37 of histone H2B are necessary and sufficient for the repression of a subset of genes in the budding yeast genome, and subsequently termed this region the H2B repression (HBR) domain [46]. This study posited a model by which the changes in gene expression that are observed upon deletion of the HBR could be due to elimination of yet to be identified PTMs that function in repression, and specifically suggest lysine 37 as a potential site of methylation [46].

**Figure 2:**
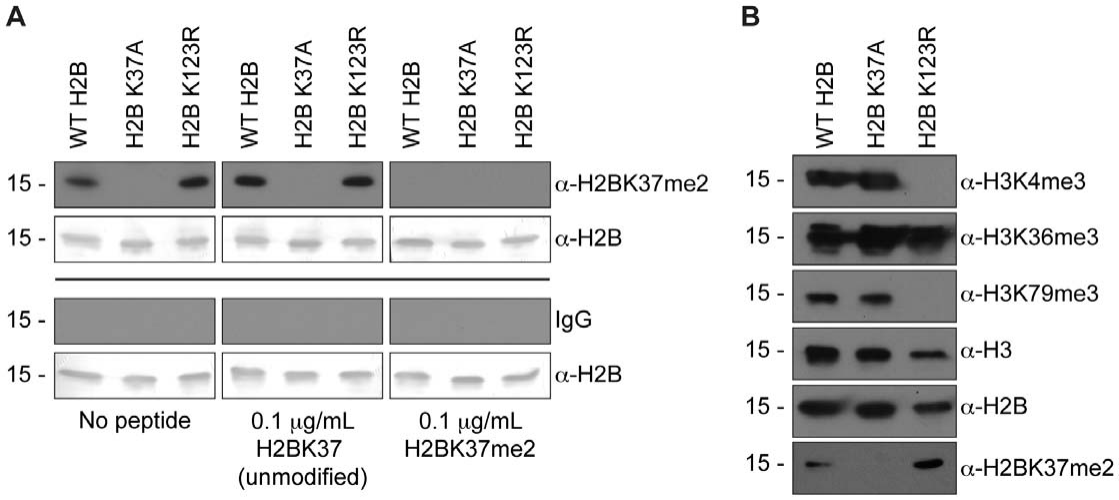
α-H2BK37me2 antibody is specific for dimethylated lysine 37 on histone H2B. (**A**) A polyclonal antibody was purified from antiserum raised by immunizing rabbits with the peptide SKARKme2ETYS-C, where me2 is dimethyl lysine. Peptide competition assay demonstrates specificity of purified α-H2BK37me2 antibody for dimethyl lysine 37 of histone H2B. Western blot analysis was completed using acid-extracted histones from strains harboring wild-type Flag-H2B (YKG001), Flag-H2B K37A (YKG007), and Flag-H2B K123R (YKG002), demonstrating that dimethylation of lysine 37 on histone H2B occurs *in vivo*, as the antibody is able to recognize this modification in wild-type and H2B K123R-derived histone samples, but not histones extracted from the Flag-H2B strain harboring a K37A mutation (No peptide controls: left column, upper panels). Preincubation of the purified antibody with H2K37me2 peptide resulted in a loss of the ∼15 kDa band in all three histone samples, whereas preincubation with unmodified H2BK37 peptide did not alter the reactivity (middle and right columns, upper panels). H2BK37me2 signal was not detectable in Western blot analysis using IgG purified from pre-immune serum (lower panels). All blots were stripped and reprobed with an α-H2B antibody to demonstrate equal loading. (**B**) Western blot analysis using modification specific antibodies indicates that mutation of lysine 37 on histone H2B does not affect methylation at other known sites of methylation in budding yeast, including histone H3 lysines 4, 36, and 79. A H2B K123R mutation abrogates methylation at H3K4 and H3K79, in agreement with previously published results [47], but does not affect H2BK37 methylation.

To validate the finding that H2B is dimethylated on lysine 37 in budding yeast, we first raised an antibody specific for this modified state in rabbit. Western blot analysis of acid-extracted wild-type histones using crude serum compared to pre-immune serum demonstrated that this mark exists *in vivo* (*data not shown*). To further corroborate this finding and characterize this novel mark, α-H2BK37me2 antibody was affinity purified from crude serum and peptide competition analysis was completed using acid-extracted wild-type H2B, H2B K37A, and H2B K123R mutant histone samples. Where affinity purified α-H2BK37me2 antibody shows a clear signal in histone samples containing wild-type H2B, mutation of lysine 37 to a non-modifiable alanine (K37A) abrogates this signal (Figure 2A, No peptide controls: left column, upper panels). Mutant H2B harboring a K123R mutation was used as a control to demonstrate specificity of this antibody for lysine 37. As a further measure of control, we showed that H2BK37me2 was not detectable in Western blot analysis using IgG purified from pre-immune serum (Figure 2A, lower panels). The affinity purified antibody is specific for dimethylation of lysine 37, as pre-incubation of the α-H2BK37me2 antibody with a dimethylated H2BK37 peptide resulted in a loss of signal in all three histone samples, but preincubation with an unmodified H2BK37 peptide did not alter reactivity (Figure 2A, middle and right columns, upper panels). Altogether, these data support the *in vivo* existence of dimethylation of histone H2B on lysine 37 and the generation of an antibody that is capable of specifically recognizing this modification.

Given that mutation of lysine 123 of histone H2B results in a loss of H2B monoubiquitylation at this site as well as a loss of methylation of histone H3 on lysines 4 and 79 [47], [48], [49], [50], [51], we sought to determine whether crosstalk existed between histone H2B lysine 37 methylation and other known sites of histone methylation in budding yeast. Western blot analysis, using acid-extracted histones from wild-type H2B and H2B K37A mutant strains, showed that the loss of H3K37 methylation did not disrupt H3K4, H3K36 or H3K79 methylation (Figure 2B). In contrast, and as a control, the H2B K123R mutant resulted in a loss of both H3K4 and H3K79 methylation, in agreement with previously published results (Figure 2B, and [47]). Finally, the H2B K123R mutation does not disrupt H2BK37 methylation (Figure 2B). Together, these results suggest that dimethylation of H2BK37 is neither affected by H2B K123 ubiquitylation nor affects the ability of additional lysine residues to be methylated.

### Elucidating the enzymes that place and remove H2BK37 methylation

**Figure 3:**
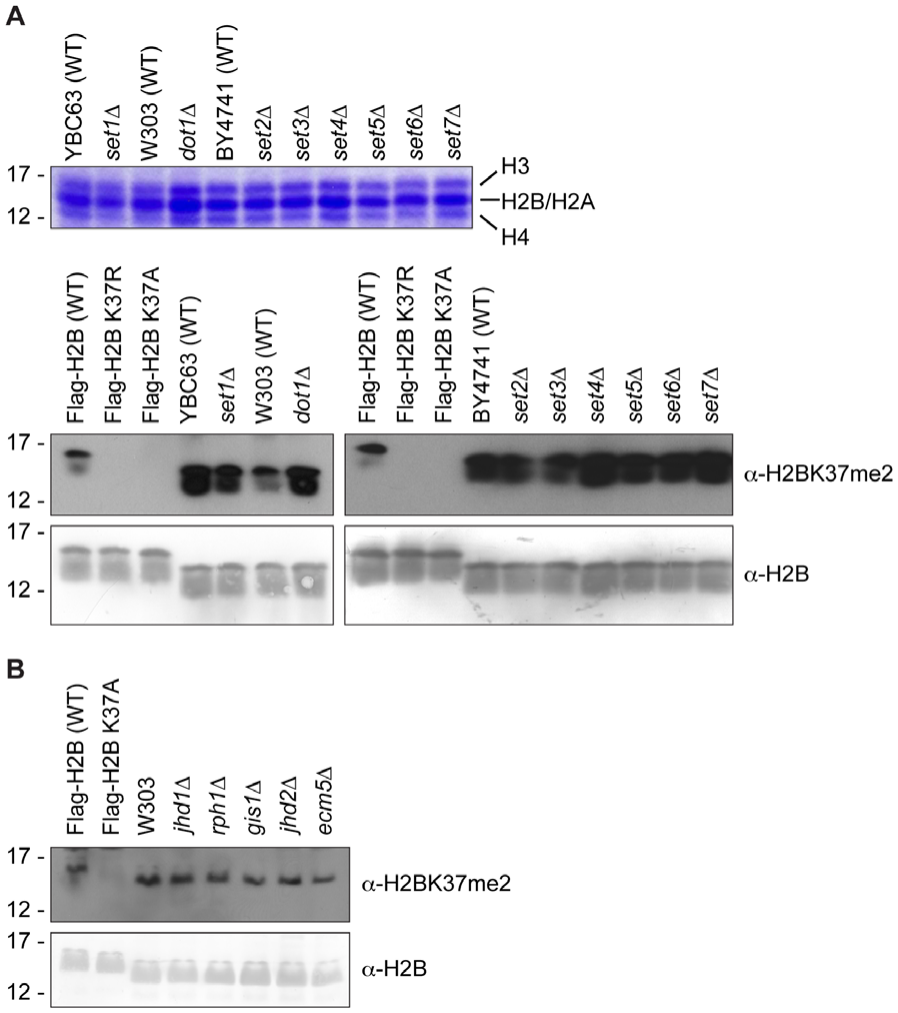
Candidate approach by Western blot analysis does not reveal the methyltransferase and demethylase responsible for H2B lysine 37 methylation. (**A**) Following validation of correct deletion of the ORF of interest and replacement with *kanMX* by genomic PCR (*data not shown*), histones were acid-extracted from candidates from the Yeast Knockout Collection (Open Biosystems), and putative histone methyltransferase activity was tested by Western blot analysis using the purified α-H2BK37me2 antibody. A Coomassie-stained gel illustrating a representative purification of histones is shown in upper panel, and representative Western blots results from the candidate screen are shown below. The blots were first probed with the α-H2BK37me2 antibody (upper) and then striped and reprobed with an α-H2B antibody (lower) to demonstrate equal loading. Histones derived from strains harboring wild-type Flag-H2B (YKG001) and Flag-H2B K37R (YKG006) or K37A (YKG007) were loaded on all gels to demonstrate loss-of-signal upon mutation of lysine 37, thereby serving as a control for antibody specificity. The presence of a Flag-tag on histone H2B results in the slight shift in electrophoretic mobility observed in the control strains, as compared to untagged H2B species in the candidate deletion strains. Deletion of candidate genes did not reveal a putative H2BK37me2 histone methyltransferase by Western blot analysis. (**B**) Histones were acid-extracted from the five JmjC-domain-containing protein deletions in *Saccharomyces cerevisiae*, and putative histone demethylase activity was analyzed by Western blot analysis using the purified α-H2BK37me2 antibody. Shown are Western blot results from the candidate screen, in which the blots were first probed with the α-H2BK37me2 antibody (upper) and then striped and reprobed with an α-H2B antibody (lower) to demonstrate equal loading. Again, histones derived from strains harboring wild-type Flag-H2B (YKG001) and Flag-H2B K37A (YKG007) were used as a control for antibody specificity, and the presence of a Flag-tag on histone H2B results in the slight shift in electrophoretic mobility observed in the control strains, as compared to untagged H2B species in the candidate deletion strains. Deletion of each individual candidate did not result in an enhanced signal, suggesting that none of these candidates function as the histone demethylase for H2BK37me2.

We next sought to identify the putative histone methyltransferase responsible for placing this mark. To this end, a candidate screen in which acid-extracted histones from individual deletion strains from the Yeast Knockout Collection (Open Biosystems) were analyzed by Western blot analysis using our α-H2BK37me2 antibody (Figure 3A). Included in the list of candidates were: the budding yeast SET-domain containing proteins; the histone lysine methyltransferase Dot1; known non-histone lysine methyltransferases; known yeast arginine methyltransferases (specific for both histone and non-histone substrates); and putative methyltransferases (Table 2). The SET domain is the catalytic domain of all identified histone lysine methyltransferases to date, with the exception of Dot1 [52]. To date, there are 12 proteins in budding yeast that harbor a SET domain (including Set1 through Set7, Rkm1 through Rkm3, and Ctm1) [53]. Of these proteins, only Set1 and Set2 have been demonstrated to function as histone lysine methyltransferases, and are specific for histone H3 lysine residues 4 and 36, respectively [54], [55], [56], [57]. Methylation of histone H3 at lysine 79 is catalyzed by Dot1, which is structurally unrelated to the other identified methyltransferases, as it lacks a SET domain altogether [58], [59]. In addition to histone lysine methyltransferases, budding yeast enzymes from the SET domain family that are capable of methylating non-histone substrates on lysine residues (namely, Ctm1, Rkm1, Rkm2, and Rkm3; [60], [61], [62], [63]) were also tested in this screen. As arginine methylation is also known to occur in budding yeast, it is possible that enzymes responsible for such modification on arginine residues could demonstrate substrate promiscuity, and thus the known arginine methyltransferases Hmt1, Rmt2, and Hsl7 [64], [65], [66] were also included in this screen. Finally, a number of annotated proteins (of both known and unknown function) predicted to function as methyltransferases based on structural predictions were also screened for activity toward histone H2B lysine 37, including the following: Trm12, Mtq1, Ylr137w, Ynl092w, Mni1, Ybr271w, Tae1, Ymr209c, Ylr063w, Ybr141c, Crg1, Yjr129c, and See1 [65], [67], [68], [69], [70], [71].

**Table 2:** Candidates screened for putative H2BK37me2 histone methyltransferase activity.

Candidate	Annotated SGD description(s)
*CRG1*	Putative S-adenosylmethionine-dependent methyltransferase; mediates cantharidin resistance
*CTM1*	Cytochrome c lysine methyltransferase; trimethylates residue 72 of apo-cytochrome c (Cyc1p) in the cytosol; not required for normal respiratory growth
*DOT1*	Nucleosomal histone H3-Lys79 methylase; methylation is required for telomeric silencing, meiotic checkpoint control, and DNA damage response
*HMT1*	Nuclear SAM-dependent mono- and asymmetric arginine dimethylating methyltransferase that modifies hnRNPs, including Npl3p and Hrp1p, affecting their activity and nuclear export; methylates U1 snRNP protein Snp1p and ribosomal protein Rps2p
*HSL7*	Protein arginine N-methyltransferase that exhibits septin and Hsl1p-dependent bud neck localization and periodic Hsl1p-dependent phosphorylation; required along with Hsl1p for bud neck recruitment, phosphorylation, and degradation of Swe1p
*MNI1*	AdoMet-dependent methyltransferase involved in a novel 3-methylhistidine modification of ribosomal protein Rpl3p; seven beta-strand MTase family member; null mutant exhibits a weak vacuolar protein sorting defect and caspofungin resistance
*MTQ1*	S-adenosylmethionine-dependent methyltransferase; methylates translational release factor Mrf1p
*RKM1*	SET-domain lysine-N-methyltransferase, catalyzes the formation of dimethyllysine residues on the large ribsomal subunit protein L23a (RPL23A and RPL23B)
*RKM2*	Ribosomal protein lysine methyltransferase, responsible for trimethylation of the lysine residue at position 3 of Rpl12Ap and Rpl12Bp
*RKM3*	Ribosomal lysine methyltransferase specific for monomethylation of Rpl42ap and Rpl42bp (lysine 40); nuclear SET domain containing protein
*RMT2*	Arginine N5 methyltransferase; methylates ribosomal protein Rpl12 (L12) on Arg67
*SEE1*	Probable lysine methyltransferase involved in the dimethylation of eEF1A (Tef1p/Tef2p); sequence similarity to S-adenosylmethionine-dependent methyltransferases of the seven beta-strand family; role in vesicular transport
*SET1*	Histone methyltransferase, subunit of the COMPASS (Set1C) complex which methylates histone H3 on lysine 4; required in transcriptional silencing near telomeres and at the silent mating type loci; contains a SET domain
*SET2*	Histone methyltransferase with a role in transcriptional elongation, methylates a lysine residue of histone H3; associates with the C-terminal domain of Rpo21p; histone methylation activity is regulated by phosphorylation status of Rpo21p
*SET3*	Defining member of the SET3 histone deacetylase complex which is a meiosis-specific repressor of sporulation genes; necessary for efficient transcription by RNAPII; one of two yeast proteins that contains both SET and PHD domains
*SET4*	Protein of unknown function, contains a SET domain
*SET5*	Zinc-finger protein of unknown function, contains one canonical and two unusual fingers in unusual arrangements; deletion enhances replication of positive-strand RNA virus
*SET6*	SET domain protein of unknown function; deletion heterozygote is sensitive to compounds that target ergosterol biosynthesis, may be involved in compound availability
*SET7/RKM4*	Ribosomal lysine methyltransferase specific for monomethylation of Rpl42ap and Rpl42bp (lysine 55); nuclear SET-domain containing protein
*TAE1*	AdoMet-dependent proline methyltransferase; catalyzes the dimethylation of ribosomal proteins Rpl12 and Rps25 at N-terminal proline residues; has a role in protein synthesis; fusion protein localizes to the cytoplasm
*TRM12*	S-adenosylmethionine-dependent methyltransferase of the seven beta-strand family; required for wybutosine formation in phenylalanine-accepting tRNA
*YBR141C*	Putative S-adenosylmethionine-dependent methyltransferase; GFP-fusion protein localizes to the nucleolus
*YBR271W*	Putative S-adenosylmethionine-dependent methyltransferase of the seven beta-strand family; GFP-fusion protein localizes to the cytoplasm; predicted to be involved in ribosome biogenesis
*YJR129C*	Putative protein of unknown function; predicted S-adenosylmethionine-dependent methyltransferase of the seven beta-strand family; GFP-fusion protein localizes to the cytoplasm
*YLR063W*	Putative S-adenosylmethionine-dependent methyltransferase; GFP-fusion protein localizes to the cytoplasm
*YLR137W*	Putative S-adenosylmethionine-dependent methyltransferase
*YMR209C*	Putative S-adenosylmethionine-dependent methyltransferase
*YNL092W*	Putative S-adenosylmethionine-dependent methyltransferase of the seven beta-strand family

We predicted that deletion of the responsible histone methyltransferase would result in a loss of signal in Western blot analysis using the α-H2BK37me2 antibody, as is observed in a parallel manner with Western blot analysis of samples derived from strains harboring individual deletions of the other known histone methyltransferases and the antibodies specific for their respective substrates. Unfortunately, all candidates screened to date (Table 2) did not give insight into the identity of the responsible methyltransferase. A loss of H2BK37me2 signal by Western blot analysis was not detected upon deletion of the individual candidates, as was observed for the control H2B K37R and H2B K37A mutants compared to their isogenic strain expressing wild-type H2B (Figure 3A, bottom). This could be due functional redundancy amongst methyltransferases, which would be masked by single gene deletions. This, however, seems unlikely, as histone methyltransferases are typically highly specific for both the lysine residue that they target as well as the degree to which they can methylate their respective substrate [72], [73]. Alternatively, another class of yet to be identified histone methyltransferases or a methyltransferase that is essential for viability could facilitate placement of this mark, in which case a candidate screen of non-essential ORFs would fail to reveal the responsible enzyme and rather an unbiased approach would have to be employed to identify the catalytic enzyme.

Recently, the JmjC domain has been identified as the catalytic domain of a family of histone demethylases [74], [75]. There are five JmjC-domain-containing proteins in budding yeast: Jhd1, Rph1, Gis1, Jhd2, and Ecm5 [76]. Jhd1, Rph1, and Jhd2 have all been demonstrated to possess histone demethylase activity, with specificity for H3K36me2/1, H3K36me3/2, and H3K4me3/2, respectively [75], [77], [78], [79], [80], [81]. We also tried a candidate approach using deletion analysis of the five JmjC-domain-containing proteins to identify a putative demethylase for this mark. Again, acid-extracted histones were analyzed by Western blot analysis using the α-H2BK37me2 antibody, with wild-type H2B and H2B K37A mutant histones serving as controls (Figure 3B). We anticipated that deletion of the putative demethylase would result in an increase in the total H2BK37me2, but deletion of the individual JmjC-domain-containing proteins did not show global changes in the level of H2BK37me2. This was not entirely surprising, as individual deletion of demethylases such as Jhd1 or Rph1 fails to show global changes in the levels of their target substrates [77], [79]. Collectively, both the methyltransferase and demethylase enzymes specifically responsible for placing and removing dimethyl marks on H2BK37 remain to be identified.

### Mutation of H2BK37 leads to no overt cellular phenotype

In parallel to identifying enzymes that catalyze the placement and removal of this methylation event, we sought to define the biological function of this mark. To this end, a number of phenotypic assays were completed using a series of strains harboring wild-type H2B, H2B K37A, H2B K37R, or H2B K123R mutant histones (in most cases, except where specifically noted, the H2B K123R mutant strain was included as a positive control). General growth at various temperatures and on various types of complete media was assessed, but both the H2B K37R and H2B K37A strains failed to show differential growth as compared to the isogenic wild-type strain. This was in contrast to the H2B K123R strain, which exhibited a slow growth phenotype at all of the temperatures and various medias assessed (*data not shown*). Examination of growth under anaerobic conditions, as well as following release from stationary phase, also failed to show a difference between the K37 mutant and wild-type histone strains (*data not shown*). Mutation of lysine 37 to either arginine or alanine also did not affect the ability of yeast cells to properly sporulate as compared to an isogenic strain expressing wild-type H2B (*data not shown*). We next posited that H2BK37me2 might be cell-cycle regulated, and therefore synchronized wild-type cells in G2/M with nocodazole and harvested cells at defined points along the cell cycle following nocodazole release. Western blot analysis of these cells at various stages of the cell cycle failed to reveal an enrichment and/or depletion of H2BK37me2 at any defined cell cycle stage (as compared to known cell-cycle regulated marks such as phosphorylation of histone H3 on serine 10 and threonine 45, which occur during mitosis and S-phase, respectively [82], [83]) (*data not shown*).

**Figure 4:**
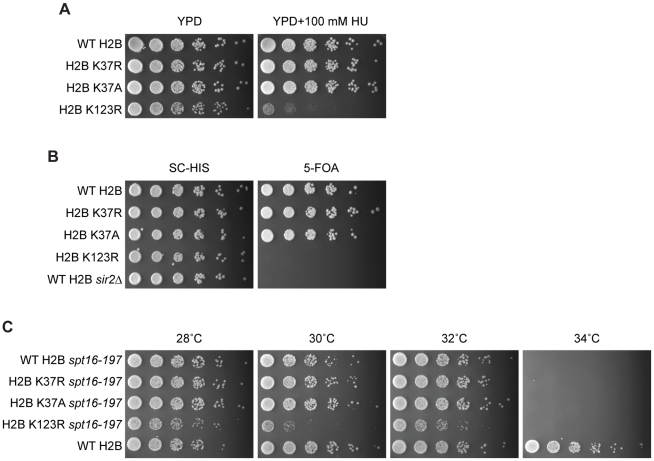
Phenotypic analysis of strains harboring H2B K37R/A mutations. (**A**) Phenotypic spotting assays indicate that cells harboring mutations at lysine 37 in histone H2B to arginine (YKG006) or alanine (YKG007) do not show sensitivity to YPD media containing 100 mM hydroxyurea (HU; a DNA damaging agent that leads to replication fork collapse), as is observed in an H2B K123R mutant strain (YKG002) [101], but rather grow similarly to yeast containing wild-type H2B (YKG001). (**B**) Telomeric silencing assay demonstrates that reporter strains harboring H2B K37R and H2B K37A mutations (YKG028 and YKG029, respectively) exhibit normal silencing like that observed for reporter strains expressing wild-type H2B (YKG027), but not strains that express an H2B K123R mutation (YZS274) or are deleted for *SIR2* (YZS275), which have known defects in telomeric silencing [48]. Growth on SC-HIS serves as a plating control, as all strains express H2B-containing plasmids carrying a *HIS3* auxotrophic marker. (**C**) Introduction of H2B K37R or K37A mutations (YKG033 and YKG034, respectively) into strains containing a temperature-sensitive allele of *SPT16* (*spt16–197*) does not affect cellular growth at the semi- and non-permissive temperatures (32°C and 34°C, respectively), as cells grow at a similar rate to those harboring wild-type H2B (YKG031). Introduction of an H2B K123R mutation (YKG032) exacerbates growth in the *spt16–197* background at the semi-permissive temperature, in agreement with previously published results [102]. The isogenic parental strain Y131 expressing wild-type *SPT16* grows phenotypically normal at the non-permissive temperature for the *spt16–197* strain.

We also performed assays to screen for phenotypes related to DNA replication and repair. To that end, wild-type H2B and the H2B K37 mutant strains were spotted on media containing the agents hydroxyurea (HU, an agent which blocks replication leading to replication fork collapse) or methyl methanesulfonate (MMS, an alkylating agent that causes DNA lesions and ultimately DNA strand breaks). However lysine 37 mutations in histone H2B did not alter cellular growth compared to an isogenic wild-type parent on media containing 0.05% MMS (*data not shown*) or 100 mM HU (Figure 4A), where cells bearing a H2B K123R mutation were sensitive to both. Moreover, to assess the ability of lysine 37 mutant strains to carry out replication, plasmid maintenance assays were completed, where the ability of a cell to replicate a reporter plasmid containing a single origin of replication and a selectable marker is measured [84]. Mutation of lysine 37 on histone H2B to either arginine or alanine did not affect the ability of yeast strains to faithfully replicate the reporter plasmid as compared to isogenic wild-type cells (*data not shown*). Taken together, the results from these screening assays suggest that histone H2B lysine 37 does not have a significant role in DNA replication or repair.

As methylation of both lysine 4 and 79 of histone H3 have been previously demonstrated to be necessary for proper telomeric silencing [48], [51], [58], [85], we next sought to determine if mutation of lysine 37 would also result in loss of telomeric silencing. To that end, H2B K37R and H2B K37A mutations were introduced into a histone H2A-H2B shuffle strain engineered to assay for defects in telomeric silencing, where expression of *URA3*, located at the left-end telomere of chromosome VII (*URA3*-TEL), is used as a readout for proper silencing [48]. If telomeric silencing properly occurs, the *URA3* gene is silenced, and cells grow normally on media containing 5-fluoroortic (5-FOA), an agent that is toxic only to cells that express *URA3*. Introduction of H2B K37R and H2B K37A mutations in *URA3*-TEL strains results in comparable growth on 5-FOA-containing media to the isogenic *URA3*-TEL strain expressing wild-type H2B (Figure 4B). This is in direct contrast to cells expressing H2B K123R or cells deleted of *SIR2*, which both fail to grow on media containing 5-FOA due to improper silencing of the *URA3* gene (Figure 4B), in agreement with previously published results [48]. Together, these data suggest that lysine 37 of histone H2B is not essential for gene silencing in yeast.

Several assays to test for transcriptional defects were also employed. Spotting assays on media containing 6-azauracil (6-AU) or mycophenolic acid (MPA), which both deplete intracellular levels of nucleotides leading to altered cellular viability when combined with mutations that affect transcriptional elongation, were completed. In both cases, strains with mutant H2B K37R or K37A grew comparably to cells with wild-type H2B, where an H2B K123R mutation resulted in a slow growth phenotype (*data not shown*). Transcription induction was also assessed by measuring the induction of *GAL1* and *GAL10* transcripts in wild-type H2B and H2B K37 mutant strains. However, gene expression analysis by reverse-transcription quantitative PCR (RT-qPCR) revealed that mutation of lysine 37 on histone H2B does not alter induction of either *GAL1* or *GAL10*, as compared to wild-type cells, supporting that this residue does not significantly contribute to transcriptional induction of these genes. Finally, we were curious to see how mutation in H2B K37 would behave in combination with mutant *SPT16*, a member of the FACT histone chaperone complex that promotes transcription elongation [86], [87], [88], [89]. Previous results have shown that the growth phenotype observed upon inactivation of *SPT16* is enhanced and suppressed by mutations in lysine residues 4 and 36 of histone H3, respectively, suggesting that FACT function is dependent upon H3K4 methylation and is opposed by H3K36 methylation [87]. We therefore introduced lysine 37 mutations into a histone H2A-H2B shuffle-strain containing a temperature-sensitive allele of *SPT16* (*spt16-197*), and cellular growth was assessed at range of temperatures. However, this analysis failed to reveal a combinatorial effect between mutation of lysine 37 on histone H2B and inactivation of *SPT16*, as H2B K37R/A *spt16-197* double mutant strains grew comparably to isogenic *spt16-197* containing wild-type H2B (Figure 4C). This is in direct opposition to a H2B K123R *spt16-197* double mutant strain, which demonstrated a synthetic effect upon inactivation of the FACT allele. These data together substantiate that methylation of lysine 37 does not appear to play a major role in transcription, as mutation of this histone residue results in no overt phenotype in all transcription-based assays completed to date.

Finally, given that Parra *et al* presented a model by which gene expression changes observed upon deletion of the HBR domain could be a consequence of eliminating a modified form of this domain [46], we sought to address whether methylation lysine 37 of H2B in particular functions in transcriptional regulation on a genomic level. To this end, gene expression changes upon mutation of lysine 37 were assessed by microarray analysis. Comparison of gene expression changes in cells expressing wild-type H2B versus a H2B K37A mutant revealed that lysine 37 does not appear to function significantly in genome-wide transcription regulation, as only 20 genes showed differential gene expression using a cutoff of a two-fold difference in expression (where two genes were upregulated (Table S1) and 18 genes were downregulated (Table S2) in a H2B K37A mutant relative to the isogenic wild-type strain). RT-qPCR analysis was able to recapitulate the microarray results of genes shown to be up- or downregulated in a H2B K37A mutant strain relative to the isogenic parent strain (Figure S1 and *data not shown*), thus validating the microarray results. However, the lack of a significant number of genes showing differential expression between wild-type and H2B K37A mutant strains indicates overall that H2BK37me2 alone does not play a major role in regulation of transcription on a genome-wide level in budding yeast.

### Methylation of H2BK37 is conserved in higher eukaryotes

**Figure 5:**
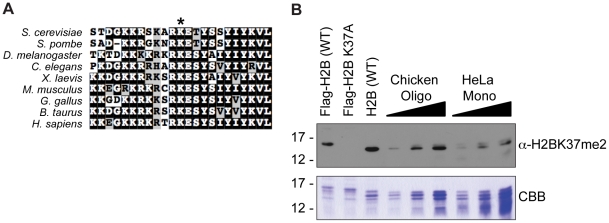
Methylation of lysine 37 of histone H2B is conserved. (**A**) Multiple sequence alignment of histone H2B from different species reveals that budding yeast histone H2B lysine 37 is conserved from yeast to humans. Sequence alignment was completed using ClustalX [103]. NCBI accession numbers are as follows: *Saccharomyces cerevisiae*: NP_010510.1; *Schizosaccharomyces pombe*: NP_588181.1; *Drosophila melanogaster*: NP_724342.1; *Caenorhabditis elegans*: NP_507031.1; *Xenopus laevis*: NP_001086753.1; *Mus musculus*: NP_783594.1; *Gallus gallus*: CAA40537.1; *Bos taurus*: DAA31692.1; *Homo sapiens*: NP_733759.1. Asterisk (*) denotes position of conserved lysine residue. (**B**) Increasing amounts of oligonucleosomes purified from chicken erythrocyte nuclei and mononucleosomes isolated from HeLa cell nuclei were run against histones extracted from yeast strains harboring wild-type Flag-H2B (YKG001), Flag-H2B K37A (YKG007), and wild-type H2B (untagged) (BY4742), as shown by Coomassie brilliant blue (CBB) staining of histone proteins electrophoresed on 15% SDS-polyacrylamide gels (lower panel). An equivalent loading of histone substrate was used for Western blot analysis using purified α-H2BK37me2 antibody (upper panel). Similar signals are detected for chicken- and human-derived histone substrates to that observed for yeast harboring wild-type H2B (either tagged or untagged), but not yeast H2B with an K37A mutation, thus demonstrating that dimethylation of histone H2B lysine 37 is conserved among species.

Sequence alignment of histone H2B from *Saccharomyces cerevisiae* against multiple species reveals that lysine 37 is conserved along evolution, despite lower sequence similarity of surrounding amino acid residues (Figure 5A). To determine if we could detect the presence of methylated lysine 37 in higher eukaryotes, we performed Western blot analysis comparing oligonucleosomes isolated from chicken erythrocyte nuclei and core histones from HeLa cell nuclei to yeast histones. Western blot analysis using the α-H2BK37me2 antibody revealed that this mark is indeed conserved in higher eukaryotes (Figure 5B), as a comparable species is observed in both the chicken and human histone samples as to histones extracted from yeast harboring wild-type, but K37A mutant, H2B. The presence of a discernable signal in samples derived from higher eukaryotic species suggests that, despite the lack of an obvious cellular phenotype in yeast to date, this mark is likely to be biologically important since it was retained during evolution.

## Discussion

To date, only six lysines residues have been identified and characterized as sites of histone methylation (namely, lysines 4, 9, 27, 36 and 79 of histone H3, and lysine 20 of histone H4) [40]. Recently, a comprehensive study employing LC-ESI MS/MS to identify PTMs of histones associated with each phase of the yeast cell cycle revealed that lysine 111 of histone H2B is also a site of histone methylation [90], in agreement with additional previously publishes results [45]. Phenotypic analyses have revealed that mutation of this lysine residue confers sensitivity to the DNA-damaging agent MMS and renders telomeric silencing defective [91], supporting the importance of this lysine residue and its methylation in chromatin function. Trimethylation of lysine 64 on histone H3 has been shown to be enriched at pericentric heterochromatin in human and mice samples, and is dynamically regulated during early development, supporting a function for this modification in the reprogramming process involved in germ cell development [92]. Additionally, methylation of histone H3 at lysine 122 has recently been reported in mice [93], and genome-wide localization patterns of methylation of lysine 5 on histone H2B have been reported in humans [41], [42]. However, the latter two sites of histone methylation are largely uncharacterized at present. It is likely that additional sites of histone lysine methylation remain to be identified, and that much remains to be discovered with regard to the complexity of histone methylation and how this PTM in particular contributes to the histone code and cellular function. That additional sites of modifications critical for normal cellular function remain to be identified thereby necessitates further investigations directed toward elucidating a complete atlas of histone PTMs.

In this manuscript, we reveal the utility of top-down MS analysis in the identification of novel histone PTMs, and report that lysine 37 of histone H2B is dimethylated in budding yeast. We also provide evidence that this modification is evolutionarily conserved. Much remains to be determined with respect to the placement and removal, regulation and biological function(s) of this mark. For example, a candidate screen employing all known lysine methyltransferases in budding yeast (both specific for histone and non-histone substrates) has revealed that the methyltransferase responsible for placement of this mark does not fall into the category of one of the previously identified methyltransferases. This suggests that either multiple methyltransferases function redundantly to methylate H2BK37, or that a novel class of methyltransferases capable of placing this mark exists. Using a similar candidate approach to screen known histone demethylases for specificity for this mark also failed to expose a demethylase specific for this mark. Given that deletion of known JmjC-domain-containing demethylases does not result in global changes in the levels of histone modifications that they have been shown to target [77], [79], it is likely that identification of the demethylase responsible for removal of lysine 37 methylation cannot be revealed by deletion analysis. Alternatively, multiple demethylases could be functionally redundant in the removal of this mark, thus making deletion analysis a less ideal assay for identification of the enzyme responsible for erasing methylation at H2BK37. It is also possible that a family of enzymes other than JmjC-domain-containing histone demethylases exists that is responsible for removal of this mark, as well as others (for example, a demethylase specific for H3K79 remains to be identified), or that there simply is not a demethylase for this mark.

*Saccharomyces cerevisiae* provides an advantageous genetic system for studying the functional consequence of loss of a specific amino acid residue (a feat that cannot be readily accomplished in higher eukaryotes [3]), thus prompting us to carry out phenotypic analysis in budding yeast. As MS analysis has revealed that H2BK37 dimethylation is a relatively abundant modification, we reasoned that mutation of lysine 37 would likely cause pleiotropic effects. However, all assays screened to date have failed to reveal a functional phenotype when lysine 37 is changed to either arginine or alanine. It is possible that this modification could function redundantly with another histone modification, in which case combinatorial mutations would be necessary to reveal the functional significance of these marks. Thus, further studies will have to be completed to determine the biological significance of this mark in chromatin.

## Materials and Methods

### Yeast strains and DNA constructs

A list of yeast strains used for these studies can be found in Table 3. Plasmids harboring wild-type or mutant histone H2B were introduced into yeast H2A-H2B shuffle strains using standard transformation [94] and shuffling [95] protocols.

**Table 3:** Yeast Strains.

Strain	Genotype	Reference/Source
FY406	*MAT* **a** *(hta1-htb1)Δ::LEU2 (hta2-htb2)Δ::TRP1 leu2Δ1 ura3-52 lys2Δ1 lys2-128δ his3Δ200 trp1Δ63 <*pSAB6 (*HTA1-HTB1 URA3*)>	[104]
YKG001	*MAT* **a** *(hta1-htb1)Δ::LEU2 (hta2-htb2)Δ::TRP1 leu2Δ1 ura3-52 lys2Δ1 lys2-128δ his3Δ200 trp1Δ63 <*pZS145 (*HTA1-Flag-HTB1 CEN HIS3*)>	[47]
YKG002	*MAT* **a** *(hta1-htb1)Δ::LEU2 (hta2-htb2)Δ::TRP1 leu2Δ1 ura3-52 lys2Δ1 lys2-128δ his3Δ200 trp1Δ63 <*pZS146 (*HTA1-Flag-htb1 (K123R) CEN HIS3*)>	[47]
YKG006	*MAT* **a** *(hta1-htb1)Δ::LEU2 (hta2-htb2)Δ::TRP1 leu2Δ1 ura3-52 lys2Δ1 lys2-128δ his3Δ200 trp1Δ63 <*pKG1 (*HTA1-Flag-htb1 (K37R) CEN HIS3*)>	This study
YKG007	*MAT* **a** *(hta1-htb1)Δ::LEU2 (hta2-htb2)Δ::TRP1 leu2Δ1 ura3-52 lys2Δ1 lys2-128δ his3Δ200 trp1Δ63 <*pKG2 (*HTA1-Flag-htb1 (K37A) CEN HIS3*)>	This study
YZS272	*MAT* **a** *ura3-1 leu2-3,112 ade2-1 trp1-1 his3-11,15 can1-100 (hta1-htb1)Δ::LEU2 (hta2-htb2)Δ <*pZS144 *(HTA1-Flag-HTB1 CEN TRP1)> URA3-TEL*	[48]
YKG027	*MAT* **a** *ura3-1 leu2-3,112 ade2-1 trp1-1 his3-11,15 can1-100 (hta1-htb1)Δ::LEU2 (hta2-htb2)Δ <*pZS145 *(HTA1-Flag-HTB1 CEN HIS3)> URA3-TEL*	This study
YKG028	*MAT* **a** *ura3-1 leu2-3,112 ade2-1 trp1-1 his3-11,15 can1-100 (hta1-htb1)Δ::LEU2 (hta2-htb2)Δ <*pKG1 *(HTA1-Flag-htb1 (K37R) CEN HIS3)> URA3-TEL*	This study
YKG029	*MAT* **a** *ura3-1 leu2-3,112 ade2-1 trp1-1 his3-11,15 can1-100 (hta1-htb1)Δ::LEU2 (hta2-htb2)Δ <*pKG2 *(HTA1-Flag-htb1 (K37A) CEN HIS3)> URA3-TEL*	This study
YZS274	*MAT* **a** *ura3-1 leu2-3,112 ade2-1 trp1-1 his3-11,15 can1-100 (hta1-htb1)Δ::LEU2 (hta2-htb2)Δ <*pZS146 *(HTA1-Flag-htb1 (K123R) CEN HIS3)> URA3-TEL*	[48]
YZS275	*MAT* **a** *ura3-1 leu2-3,112 ade2-1 trp1-1 his3-11,15 can1-100 (hta1-htb1)Δ::LEU2 (hta2-htb2)Δ <*pZS145 *(HTA1-Flag-HTB1 CEN HIS3)> URA3-TEL sir2Δ::TRP1*	[48]
YZS276	*MAT* **a** *(hta1-htb1)Δ::LEU2 (hta2-htb2)Δ leu2-3,112 his3-11,15 trp1-1 ura3-1 ade2-1 can 1-100 <*pZS145 *(HTA1-Flag-HTB1 CEN HIS3)>*	[48]
YZS277	*MAT* **a** *(hta1-htb1)Δ::LEU2 (hta2-htb2)Δ leu2-3,112 his3-11,15 trp1-1 ura3-1 ade2-1 can 1-100 <*pZS146 *(HTA1-Flag-htb1 (K123R) CEN HIS3)>*	[48]
Y131	*MAT* **a** *(hta1-htb1)Δ::LEU2 (hta2-htb2)Δ leu2-3,112 trp1-1 ura3-1 ade2-1 can1-100 his3-11,15* <pRS426 (*HTA1-HTB1 URA3 2 µm*)>	[105]
YCH278	*MAT* **a** *(hta1-htb1)Δ::LEU2 (hta2-htb2)Δ leu2-3,112 trp1-1 ura3-1 ade2-1 can1-100 his3-11,15 spt16::kanMX <*pRS426 *(HTA-HTB URA3 2 µm)> <*pBM46*-spt16-197>*	[102]
YKG031	*MAT* **a** *(hta1-htb1)Δ::LEU2 (hta2-htb2)Δ leu2-3,112 trp1-1 ura3-1 ade2-1 can1-100 his3-11,15 spt16::kanMX <*pZS145 *(HTA1-Flag-HTB1 CEN HIS3)> <*pBM46*-spt16-197>*	This study
YKG032	*MAT* **a** *(hta1-htb1)Δ::LEU2 (hta2-htb2)Δ leu2-3,112 trp1-1 ura3-1 ade2-1 can1-100 his3-11,15 spt16::kanMX <*pZS146 *(HTA1-Flag-htb1 (K123R) CEN HIS3)> <*pBM46*-spt16-197>*	This study
YKG033	*MAT* **a** *(hta1-htb1)Δ::LEU2 (hta2-htb2)Δ leu2-3,112 trp1-1 ura3-1 ade2-1 can1-100 his3-11,15 spt16::kanMX <*pKG1 *(HTA1-Flag-htb1 (K37R) CEN HIS3)> <*pBM46*-spt16-197>*	This study
YKG034	*MAT* **a** (*hta1-htb1)Δ::LEU2 (hta2-htb2)Δ leu2-3,112 trp1-1 ura3-1 ade2-1 can1-100 his3-11,15 spt16::kanMX <*pKG2 *(HTA1-Flag-htb1 (K37A) CEN HIS3)> <*pBM46*-spt16-197>*	This study
YMP001	*MAT*α *leu2-3,112 trp1-1 can1-100 ura3-1 ade2-1 his3-11,15 rad5-535 HTZ1::myc/7xHis*	This study
YBC63	*MAT*α *lys2-128 leu2Δ ura3-52 trp1Δ63 his3Δ200*	[106]
YBC1236	*MAT*α *lys2-128 leu2Δ ura3-52 trp1Δ63 his3Δ200 set1Δ::HIS3MX6*	[106]
DY2390 (W303)	*MAT*α *ade2 can1 his3 leu2 lys2 trp1 ura3*	[107]
YAR005	*MAT*α *ade2 can1 his3 leu2 lys2 trp1 ura3 rph1Δ::kanMX*	This study
YAR007	*MAT*α *ade2 can1 his3 leu2 lys2 trp1 ura3 jhd1Δ::kanMX*	This study
YAR009	*MAT*α *ade2 can1 his3 leu2 lys2 trp1 ura3 gis1Δ::kanMX*	This study
YAR011	*MAT*α *ade2 can1 his3 leu2 lys2 trp1 ura3 jhd2Δ::kanMX*	This study
YAR013	*MAT*α *ade2 can1 his3 leu2 lys2 trp1 ura3 ecm5Δ::kanMX*	This study
YNL037	*MAT*α *ade2 can1 his3 leu2 lys2 trp1 ura3 dot1Δ::kanMX*	This study
BY4741	*MAT* **a** *his3Δ1 leu2Δ0 met15Δ0 ura3Δ0*	Open Biosystems
BY4742	*MAT*α *his3Δ1 leu2Δ0 lys2Δ0 ura3Δ0*	Open Biosystems

The following deletion strains used for candidate screening are from the Yeast Knockout Collection in the BY4741 background (Open Biosystems): *crg1Δ::kanMX*, *ctm1Δ::kanMX*, *htm1Δ::kanMX*, *mni1Δ::kanMX*, *mtq1Δ::kanMX*, *rkm1Δ::kanMX*, *rkm2Δ::kanMX*, *rkm3Δ::kanMX*, *rmt2Δ::kanMX*, *see1Δ::kanMX*, *set2Δ::kanMX*, *set3Δ::kanMX*, *set4Δ::kanMX*, *set5Δ::kanMX*, *set6Δ::kanMX*, *set7Δ::kanMX*, *tae1Δ::kanMX*, *trm12Δ::kanMX*, *ybr141cΔ::kanMX*, *ybr271wΔ::kanMX*, *yjr129cΔ::kanMX*, *ylr063wΔ::kanMX*, *ylr137wΔ::kanMX*, *ymr209cΔ::kanMX*, *ynl092wΔ::kanMX*. The following deletion strain used for candidate screening is from the Yeast Knockout Collection in the BY4742 background (Open Biosystems): *hsl7Δ::kanMX*.

The plasmids pZS145 (*HTA1*-Flag-*HTB1 CEN HIS3*) and pZS146 (*HTA1*-Flag-*htb1* (*K123R*) *CEN HIS3*) were isolated from the strains YZS276 and YZS277, respectively, obtained from Z.W. Sun [48]. The plasmids pKG1 (*HTA1*-Flag-*htb1* (*K37R*) *CEN HIS3*) and pKG2 (*HTA1*-Flag-*htb1* (*K37A*) *CEN HIS3*) were derived from site-directed mutagenesis of pZS145 [48] using the QuikChange II Site-Directed Mutagenesis kit (Stratagene). The accuracy of all constructs was verified by DNA sequencing.

### Histone acid extraction

Histones were extracted from yeast nuclei using a standard acid extraction method [96]. Briefly, 250 mL cultures were grown at 30°C to an OD_600_ approximately equal to 1.5. Cells were collected by centrifugation at 2700×*g* for 5 minutes, washed once with sterile water, and collected again by centrifugation. Cells were resuspended in 7.5 mL Solution 1 [0.1 mM Tris-Cl (pH 9.4), 10 mM DTT], and then incubated at 30°C for 15 minutes with shaking at 100 rpm. Cells were collected by centrifugation at 2700×*g* for 5 minutes, washed in 15 mL Solution 2 [1.2 M sorbitol, 20 mM HEPES-OH (pH 7.4)], and pelleted again. Cells were resuspended in 15 mL Solution 2 containing Zymolyase 20T at a final concentration of 0.2 mg/mL, and were then incubated at 30°C with shaking at 100 rpm until spheroplasting was greater than 90% (as determined by measuring the OD_600_ of 10 µL sample in 1 mL 1% SDS; typically 45–50 minutes), at which point 15 mL ice-cold Solution 3 [1.2 M sorbitol, 20 mM PIPES-OH (pH 6.8), 1 mM MgCl_2_] was added. Cells were pelleted again at 1300×*g* for 5 minutes 4°C. Pellets were resuspended in 7.5 mL ice-cold Solution 4 [250 mM sucrose, 60 mM KCl, 14 mM NaCl, 5 mM MgCl_2_, 1 mM CaCl_2_, 15 mM MES (pH 6.6), 1 mM PMSF, 0.8% TritonX-100], incubated on ice for 20 minutes, and spun at 1700×g for 5 minutes at 4°C. Nuclei isolation in Solution 4 was completed a total of three times. Nuclei were washed three times in 12.5 mL Wash 1 [10 mM Tris-Cl (pH 8.0), 0.5% NP-40, 75 mM NaCl, 1 mM PMSF] for 15 minutes on ice for the first two washes, and 5 minutes on ice for the third wash, followed by two washes in 12.5 mL Wash 2 [10 mM Tris-Cl (pH 8.0), 400 mM NaCl, 1 mM PMSF] for 10 minutes on ice for the first wash, and centrifuged immediately following the second resuspension. Histones were extracted in 1.5 mL 0.4 N H_2_SO_4_ with incubation on ice for 30 minutes, with occasional vortexing. Debris was pelleted by centrifugation at 10,000×*g*. Histone proteins were precipitated from the supernatent by addition of 100% TCA to a final concentration of 20% with incubation on ice for 30 minutes. Histone proteins were pelleted at 15,000×*g*. Pellets were washed once with acetone containing 1% HCl, and once with acetone. After being air-dried, histone proteins were resuspended in 300 µL 10 mM Tris-Cl (pH 8.0).

### Reverse-phase HPLC purification of histone proteins

Following sulfuric acid extraction, histones derived from the strain YMP001 were subject to RP-HPLC isolation. Gradient conditions used for histone isolation were adapted from conditions previously described [97]. Briefly, proteins from sulfuric acid extracts were injected onto a Zorbex C-18 column with a pore size of 3.5 µm using an Agilent 1100 series RP-HPLC (Agilent, Santa Clara CA). The column was washed and prepared using the following method: 5–35% Acetonitrile (CH_3_CN) with 0.1% Trifluoroacetic acid (TFA) for 5 minutes followed by 35% CH_3_CN/0.1% TFA for 10 minutes. Histones were separated using the following gradient: 35%–60% CH_3_CN/0.1%TFA for 30 minutes [98]. Protein elution was monitored by UV absorption at 220 nm. Fractions containing histone H2B were determined by Western blot analysis using an α-H2B antibody (Active Motif, Cat. No. 39237).

### μESI-FTICR-MS analysis

#### MS Conditions

Acquisition of MS spectra was performed using a hybrid Qe-Fourier Transform Ion Cyclotron Resonance - Mass Spectrometer, equipped with a 12.0 Tesla actively shielded magnet (Apex Qe-FTICR-MS, 12.0 T AS, Bruker Daltonics, Billerica, MA, USA), and an Apollo II microelectrospray (μESI) source. The voltages on μESI spray capillary, spray shield, capillary exit, deflector, ion funnel and skimmer were set at +4.2 kV, +3.6 kV, +340 V, +310 V, +185 V and +25 V, respectively. The temperature of the μESI source was maintained at 120°C. Desolvation was carried out using a nebulization gas flow (2.0 bar) and a countercurrent drying gas flow (4.0 L/s). Histone H2B samples were prepared by resuspending lyophilized RP-HPLC fractions containing H2B in a mixture of acetonitrile/water/acetic acid (49.0∶49.0∶2.0 v/v/v) at a concentration of 0.1–0.2 µg/µL, directly infused with a syringe pump (Harvard Apparatus, Holliston, MA, USA) and a 100-µL syringe (Hamilton, Reno, NV, USA), and electrosprayed at an infusion flow rate of 90 µL/hr. Before transfer, ion packets were accumulated inside the collision cell for a duration of 0.5–1.0 seconds. 100 MS scans per spectrum were acquired in the ICR cell with a resolution of 580,000 at m/z 400 Da.

#### MS/MS Conditions

FTICR-ECD MS/MS method was employed to fragment histone H2B. Precursor ions were isolated with a quadrupole (Q1) and subjected to ICR cell directly. The isolation window width was 2.0 Da. Low energy electrons were generated by the heated hollow dispenser cathode with a bias voltage of −2.5 V. ECD lens voltage was set at +15.0 V. The electrons, produced by the hollow dispenser cathode (operated at 1.7 A), were pulsed into the ICR cell with a length of 3.0 ms, which led to fragmentation of the ions that were already trapped in the ICR cell. To maximize the ion population before irradiation, the ICR cell was filled with 1–5 iterations of ion accumulation from the external collision cell [27]. 100 MS/MS scans per spectrum were acquired with a resolution of 580,000 at m/z 400 Da.

### α-H2BK37me2 antibody production and antibody affinity purification

A synthetic peptide containing H2B sequence from 33 to 41, in which lysine 37 was dimethylated, was conjugated to keyhole limpet hemocyanin via a C-terminal cysteine in the peptide and was used to immunize rabbits (Pocono Rabbit Farm and Laboratory Inc.). The α-H2BK37me2 antibody was affinity purified from serum. Briefly, equilibrated Affigel-10 (BIORAD) was incubated with the peptide SKARKme2ETYS-C (where me2 is dimethyl lysine) in PBS for 2 hr at 4°C. Unbound peptide was removed, and the peptide-bound resin was blocked with 0.2 M ethanolamine (pH 8.0) for 2 hr at 4°C. After washing with 1 M NaCl and PBS, the blocked peptide-bound resin was incubated with serum for 3 hr at room temperature with rotation. The flow-through was collected, and the resin was washed with 0.5 M NaCl followed by PBS. Antibody was eluted with 0.1 M glycine (pH 3.0) at one-half column volume/fraction, and 1/10 (v/v) 1 M Tris-Cl (pH 8.0) was added to neutralize the pH. Purity of antibody fractions were analyzed on 12% SDS-polyacrylamide gels followed by Coomassie-staining, allowing for pooling of peak antibody fractions.

IgG was purified from pre-immune serum. Briefly, Protein A beads (GE Healthcare) pre-equilibrated with Tris-salt buffer [100 mM Tris-Cl (pH 7.95), 135 mM NaCl] were incubated with pre-immune serum for 2 hr at room temperature with rotation. The flow-through was collected, and the column was washed with Tris-salt buffer, followed by 10 mM Tris-Cl (pH 7.95). IgG was eluted with 0.1 M glycine (pH 3.0) at one-half column volume/fraction, and 1/10 (v/v) 1 M Tris-Cl (pH 8.0) was added to neutralize the pH. Purity of IgG fractions were analyzed on 12% SDS-polyacrylamide gels followed by Coomassie-staining, allowing for pooling of peak IgG fractions.

### Western blot analysis and peptide competition assay

Histone samples were run on 15% SDS-polyacrylamide gels, which were transferred to PVDF membranes (Pall Corporation) using a semi-dry apparatus (Hoefer) and Towbin buffer. Membranes were blotted using standard techniques, and probed with the antibodies at the following dilutions: α-H3 (Active Motif, Cat. No. 39163; 1∶5000), α-H2BK37me2 (PRF&L, generated in this study; 1∶2000), α-H3K4me3 (Active Motif, Cat. No. 39159; 1∶10,000), α-H3K36me3 (Abcam, Cat. No. ab9050; 1∶2000), α-H3K79me3 (Abcam, Cat. No. ab2621; 1∶2000), or α-H2B (Active Motif, Cat. No. 39237; 1∶10,000).

For peptide competition assays to demonstrate the specificity of purified α-H2BK37me2 antibody for H2BK37me2, purified IgG or α-H2BK37me2 antibody was pre-incubated with no peptide, a H2K37 peptide (SKARKETYS-C) or a H2K37me2 peptide (SKARKme2ETYS-C, where me2 is dimethyl lysine) at a final peptide concentration of 0.1 µg/mL for 1.5 hr at room temperature prior to incubation of PVDF membranes with primary antibody followed by standard Western blot analysis.

### RNA isolation, microarray and RT-qPCR mRNA analyses

Yeast cultures were grown at 30°C in YPD simultaneously in triplicate to an OD_600_ of approximately 1.0. Ten OD_600_ units of cells were collected, washed once with water, and pellets were flash frozen in liquid nitrogen. Total RNA was isolated using the hot acidic phenol-chloroform method [99]. Briefly, cell pellets were resuspended in 400 µL TES solution [10 mM Tris-Cl (pH 7.5), 10 mM EDTA, 0.5% SDS], to which 400 µL acidic phenol-chloroform (Ambion) was added. Samples were vortexed vigorously, incubated at 65°C for 1 hour with occasional vortexing, and then placed on ice for 5 min. The aqueous layer was back-extracted once with acidic phenol-chloroform and once with chloroform. Following back-extraction with chloroform, RNA was precipitated using a standard ethanol precipitation protocol, and resuspended in RNase-free water. RNA was cleaned up using an RNeasy Mini Kit (QIAGEN), and RNA quality was determined using an Agilent Bioanalyzer.

Biotinylated-cRNA was generated using the MessageAmp™II-Biotin Enhanced Kit (Ambion) and was hybridized to Yeast Genome 2.0 arrays (Affymetrix), following manufacturer's protocol. Briefly, hybridizations were completed for 16 hr at 45°C at 60 rpm in a GeneChip Hybridization Oven 640. Arrays were washed and stained using the GeneChip Fluidics Station 450, and were scanned with the GeneChip Scanner 3000 7G Plus Scanner with Autoloader. Microarray hybridization and analysis was completed at the University of North Carolina at Chapel Hill Functional Genomics Core Facility.

For real-time quantitative PCR (qPCR) gene expression analysis, following treatment of isolated RNA with DNA-*free* (Ambion) and RNA clean-up using an RNeasy Mini Kit (QIAGEN), first-strand cDNA was generated from total RNA using the Improm-II Reverse Transcription System (Promega). PCR reactions using 1/20 of total cDNA as template were completed using primers specific to the indicated genes. Primers used are as follows: *ACT1* Forward: GAGGTTGCTGCTTTGGTTATTGA, Reverse: ACCGGCTTTACACATACCAGAAC. *AQR1* 5′ Forward: GCTTTGAGGCAGTTGGAAAA, 5′ Reverse: CACCGCTAACTGTGGGAGAT; *AQR1* 3′ Forward: TGGGTTCCTTCTTCACAGGT, 3′ Reverse: CTCTGCGTCTTGTGGAATCA. *FMP43* 5′ Forward: ATTAGCGACGGCACTGATTT, 5′ Reverse: CAGTGCAACCCAGGAAAAA; *FMP43* 3′ Forward: GGATACGGAACGGTGATTCT, 3′ Reverse: TCATCGATGTGGATGCAGTT. PCR reactions were carried out in triplicate for qPCR analysis using SYBR GreenER qPCR master mix (Invitrogen) and the Applied Biosystems 7900HT Fast Real-Time PCR system.

### Microarray data

All microarray data is MIAME compliant. Raw data generated from these studies have been deposited into the MIAME compliant database Gene Expression Omnibus (NCBI, http://www.ncbi.nlm.nih.gov/geo/) and are accessible through GEO series accession number GSE24380.

### Phenotypic spotting assays

To assay for growth in phenotypic spotting assays, five-fold serial dilutions of saturated overnight yeast cultures grown in YPD medium, or in synthetic complete medium supplemented as appropriate for plasmid selection, were plated onto appropriate media at a starting OD_600_ of 0.5. Growth on plates was imaged after 2–4 days of incubation at 30°C, unless temperature is otherwise indicated.

## Supporting Information

Figure S1 **RT-qPCR analysis recapitulates microarray results of gene expression changes upon mutation of H2B lysine 37.** Yeast cells harboring wild-type H2B (YKG001) or a H2B K37A mutation (YKG007) were grown to mid-log phase, and RNA samples were isolated. The expression of genes identified as up- or downregulated upon mutation of lysine 37 by microarray analysis was verified by RT-quantitative real time PCR analysis (RT-qPCR). Representative RT-qPCR analysis is shown for *AQR1* and *FMP43*, which were up- and downregulated, respectively, in yeast cells harboring the H2B K37A mutation relative to wild-type H2B according to microarray analysis. Gene expression was normalized against actin (*ACT1*).
(TIF)

Table S1 **Genes that are upregulated at least two-fold in H2B K37A mutant cells.**
(DOC)

Table S2 **Genes that are downregulated at least two-fold in H2B K37A mutant cells.**
(DOC)
