# Supplementary figures and images for: Identification of Lysine 37 of Histone H2B as a Novel Site of Methylation
Source: PLoS One. 2011 Jan 13;6(1):e16244. doi: 10.1371/journal.pone.0016244 (PMC3020972; doi:10.1371/journal.pone.0016244)

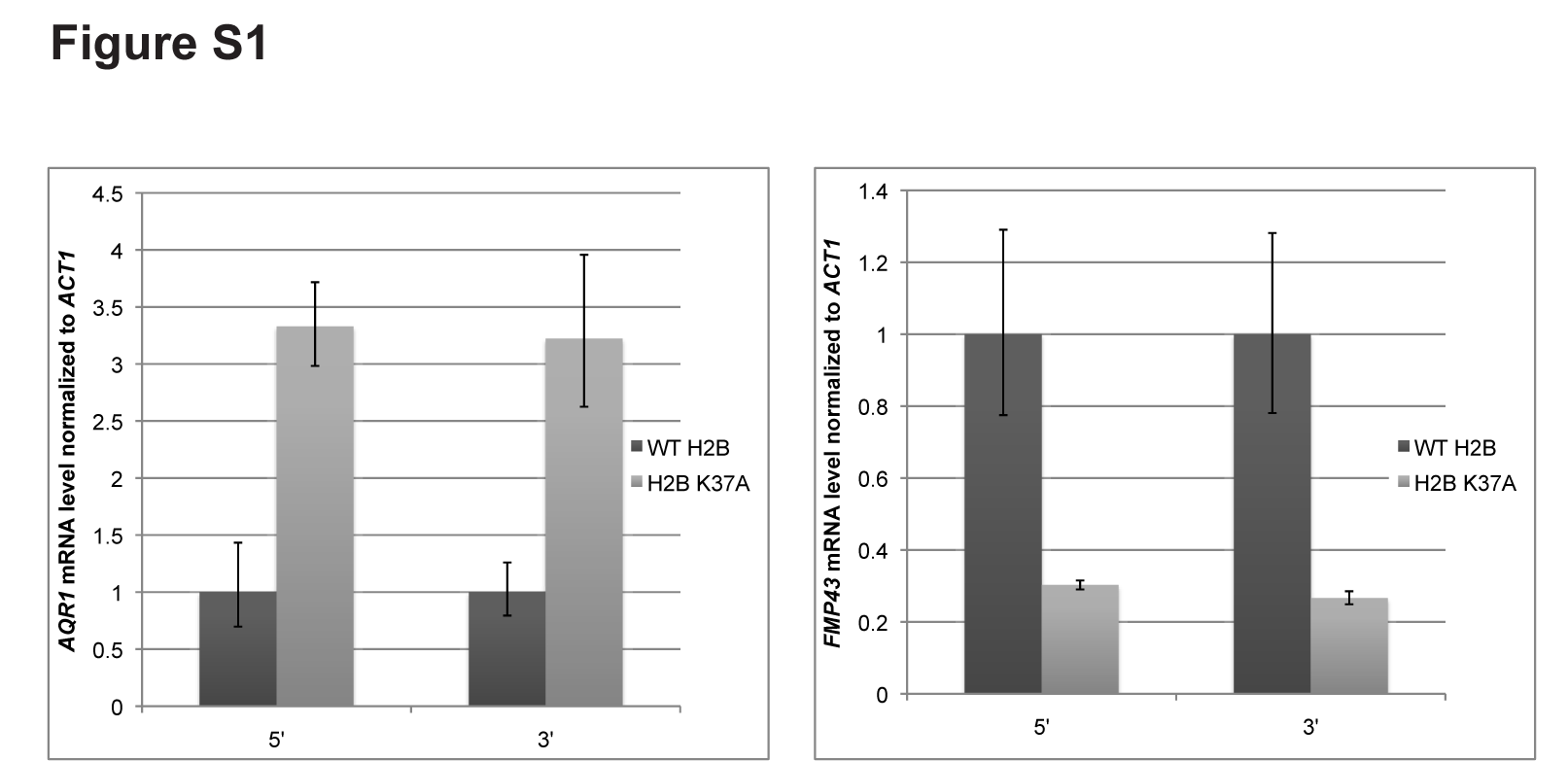

Supplement: Figure S1 — RT-qPCR analysis recapitulates microarray results of gene expression changes upon mutation of H2B lysine 37. Yeast cells harboring wild-type H2B (YKG001) or a H2B K37A mutation (YKG007) were grown to mid-log phase, and RNA samples were isolated. The expression of genes identified as up- or downregulated upon mutation of lysine 37 by microarray analysis was verified by RT-quantitative real time PCR analysis (RT-qPCR). Representative RT-qPCR analysis is shown for AQR1 and FMP43, which were up- and downregulated, respectively, in yeast cells harboring the H2B K37A mutation relative to wild-type H2B according to microarray analysis. Gene expression was normalized against actin (ACT1). (TIF) [file pone.0016244.s001.tif]
