# Supplementary material for: Identification of Lysine 37 of Histone H2B as a Novel Site of Methylation
Source: PLoS One. 2011 Jan 13;6(1):e16244. doi: 10.1371/journal.pone.0016244 (PMC3020972; doi:10.1371/journal.pone.0016244)
Supplement: Table S1 — Genes that are upregulated at least two-fold in H2B K37A mutant cells. (DOC) [file pone.0016244.s002.doc]

**Table S1 | Genes that are upregulated at least two-fold in H2B K37A mutant cells**

| **Candidate** | **Name** | **Fold Change*** | **Annotated SGD description(s)** |
| --- | --- | --- | --- |
| YNL065W | *AQR1* | 2.50 | Plasma membrane multidrug transporter of the major facilitator superfamily, confers resistance to short-chain monocarboxylic acids and quinidine; involved in the excretion of excess amino acids |
| YGR079W | *---* | 2.30 | Putative protein of unknown function; YGR079W is not an essential gene |

*K37A:WT
