# Supplementary material for: Identification of Lysine 37 of Histone H2B as a Novel Site of Methylation
Source: PLoS One. 2011 Jan 13;6(1):e16244. doi: 10.1371/journal.pone.0016244 (PMC3020972; doi:10.1371/journal.pone.0016244)
Supplement: Table S2 — Genes that are downregulated at least two-fold in H2B K37A mutant cells. (DOC) [file pone.0016244.s003.doc]

**Table S2 | Genes that are downregulated at least two-fold in H2B K37A mutant cells**

| **Candidate** | **Name** | **Fold Change*** | **Annotated SGD description(s)** |
| --- | --- | --- | --- |
| YHR209W | *CRG1* | 0.49 | Putative S-adenosylmethionine-dependent methyltransferase; mediates cantharidin resistance |
| YKL163W | *PIR3* | 0.49 | O-glycosylated covalently-bound cell wall protein required for cell wall stability; expression is cell cycle regulated, peaking in M/G1 and also subject to regulation by the cell integrity pathway |
| YEL011W | *GLC3* | 0.48 | Glycogen branching enzyme, involved in glycogen accumulation; GFP-fusion protein localizes to the cytoplasm in a punctate pattern |
| YOR028C | *CIN5* | 0.48 | Basic leucine zipper (bZIP) transcription factor of the yAP-1 family, mediates pleiotropic drug resistance and salt tolerance; nuclearly localized under oxidative stress and sequestered in the cytoplasm by Lot6p under reducing conditions |
| YHR184W | *SSP1* | 0.48 | Protein involved in the control of meiotic nuclear division and coordination of meiosis with spore formation; transcription is induced midway through meiosis |
| YMR101C | *SRT1* | 0.48 | Cis-prenyltransferase involved in synthesis of long-chain dolichols (19-22 isoprene units; as opposed to Rer2p which synthesizes shorter-chain dolichols); localizes to lipid bodies; transcription is induced during stationary phase |
| YBR147W | *RTC2* | 0.47 | Protein of unknown function; identified in a screen for mutants with decreased levels of rDNA transcription; detected in highly purified mitochondria; null mutant suppresses *cdc13-1*; similar to a G-protein coupled receptor from *S. pombe* |
| YDL169C | *UGX2* | 0.47 | Protein of unknown function, transcript accumulates in response to any combination of stress conditions |
| YDL079C | *MRK1* | 0.47 | Glycogen synthase kinase 3 (GSK-3) homolog; one of four GSK-3 homologs in *S. cerevisiae* that function to activate Msn2p-dependent transcription of stress responsive genes and that function in protein degradation |
| YMR206W | *---* | 0.46 | Putative protein of unknown function; YMR206W is not an essential gene |
| YGL158W | *RCK1* | 0.45 | Protein kinase involved in the response to oxidative stress; identified as suppressor of *S. pombe* cell cycle checkpoint mutations |
| YOR178C | *GAC1* | 0.45 | Regulatory subunit for Glc7p type-1 protein phosphatase (PP1), tethers Glc7p to Gsy2p glycogen synthase, binds Hsf1p heat shock transcription factor, required for induction of some HSF-regulated genes under heat shock |
| YFL052W | *ROP1* | 0.44 | Putative zinc cluster protein that contains a DNA binding domain; null mutant sensitive to calcofluor white, low osmolarity and heat, suggesting a role for YFL052Wp in cell wall integrity |
| YDR277C | *MTH1* | 0.44 | Negative regulator of the glucose-sensing signal transduction pathway, required for repression of transcription by Rgt1p; interacts with Rgt1p and the Snf3p and Rgt2p glucose sensors; phosphorylated by Yck1p, triggering Mth1p degradation |
| YGR243W | *FMP43* | 0.43 | Putative protein of unknown function; expression regulated by osmotic and alkaline stresses; the authentic, non-tagged protein is detected in highly purified mitochondria in high-throughput studies |
| YBR299W | *MAL32* | 0.43 | Maltase (alpha-D-glucosidase), inducible protein involved in maltose catabolism; encoded in the MAL3 complex locus; functional in genomic reference strain S288C; hydrolyzes the disaccharides maltose, turanose, maltotriose, and sucrose |
| YMR280C | *CAT8* | 0.42 | Zinc cluster transcriptional activator necessary for derepression of a variety of genes under non-fermentative growth conditions, active after diauxic shift, binds carbon source responsive elements |
| YIL057C | *RGI2* | 0.37 | Putative protein of unknown function; expression induced under carbon limitation and repressed under high glucose |

*K37A:WT
